# Defining the *in vivo* characteristics of acute myeloid leukemia cells behavior by intravital imaging

**DOI:** 10.1111/imcb.12216

**Published:** 2018-12-13

**Authors:** Delfim Duarte, Saoirse Amarteifio, Heather Ang, Isabella Y Kong, Nicola Ruivo, Gunnar Pruessner, Edwin D Hawkins, Cristina Lo Celso

**Affiliations:** ^1^ Department of Life Sciences Sir Alexander Fleming Building Imperial College London London SW7 2AZ UK; ^2^ Instituto de Investigação e Inovação em Saúde (i3S) University of Porto Porto 4200‐135 Portugal; ^3^ Department of Biomedicine Faculty of Medicine University of Porto Porto 4200‐319 Portugal; ^4^ Department of Onco‐Hematology Portuguese Institute of Oncology (IPO)‐Porto Porto 4200‐072 Portugal; ^5^ Department of Mathematics Huxley Building Imperial College London London SW7 2AZ UK; ^6^ The Walter and Eliza Hall Institute of Medical Research Melbourne VIC 3052 Australia; ^7^ Department of Medical Biology The University of Melbourne Parkville VIC 3010 Australia; ^8^ The Francis Crick Institute London WC2A 3LY UK

**Keywords:** Acute myeloid leukemia cells, chemokines, intravital imaging, lymphoblastic leukemia

## Abstract

The majority of acute myeloid leukemia (AML) patients have a poor response to conventional chemotherapy. The survival of chemoresistant cells is thought to depend on leukemia‐bone marrow (BM) microenvironment interactions, which are not well understood. The CXCL12/CXCR4 axis has been proposed to support AML growth but was not studied at the single AML cell level. We recently showed that T‐cell acute lymphoblastic leukemia (T‐ALL) cells are highly motile in the BM; however, the characteristics of AML cell migration within the BM remain undefined. Here, we characterize the *in vivo* migratory behavior of AML cells and their response to chemotherapy and CXCR4 antagonism, using high‐resolution 2‐photon and confocal intravital microscopy of mouse calvarium BM and the well‐established MLL‐AF9‐driven AML mouse model. We used the Notch1‐driven T‐ALL model as a benchmark comparison and AMD3100 for CXCR4 antagonism experiments. We show that AML cells are migratory, and in contrast with T‐ALL, chemoresistant AML cells become less motile. Moreover, and in contrast with T‐ALL, the *in vivo* exploratory behavior of expanding and chemoresistant AML cells is unaffected by AMD3100. These results expand our understanding of AML cells‐BM microenvironment interactions, highlighting unique traits of leukemia of different lineages.

## Introduction

Acute myeloid leukemias (AML) are aggressive myeloid lineage leukemias with a poor prognosis. This is due to poor responses to current chemotherapy regimens.^1^ Therefore, there is a pressing need to understand how drug resistance develops in AML so novel therapeutic interventions can be investigated. A well‐established hypothesis is that leukemic cells depend on protective bone marrow (BM) niches to expand and survive.^2^ Of particular interest are protective niches that may facilitate the survival of minimal residual disease during chemotherapy, the leading cause of leukemia relapse.^2^ However, there is still little information on the dynamics of AML cells themselves *in vivo* that support this hypothesis. We, and others, have reported AML to be associated with endosteal niches^2^, ^3^, ^4^, but the dynamics of AML interactions with the BM microenvironment and whether this process is linked to AML chemoresistance and minimal residual disease remains unanswered. Using intravital microscopy, we recently showed that Notch1‐driven T‐cell acute lymphoblastic leukemia (T‐ALL) cells (and particularly, chemoresistant clones) are highly motile with behavior that is seemingly independent from specific microenvironments.^5^ The role of cell motility and how this is directed by leukemia‐microenvironment interactions in AML pathogenesis has not yet been investigated.^6^

CXCL12 is abundantly secreted in the marrow stroma and binds to the receptor CXCR4. CXCL12 is fundamental for the retention of CXCR4‐expressing cells in the BM. We previously showed that up‐regulation of CXCR4 is associated with increased engraftment and motility of hematopoietic stem cells within the BM microenvironment.^7^ CXCR4 inhibition prolongs the survival of T‐ALL burdened mice,^8^ and promotes mobilization and apoptosis of AML cells.^9^, ^10^, ^11^ CXCR4 antagonists in combination with chemotherapy have been tested in phase 1/2 clinical trials in relapsed and refractory AML (reviewed in Cho *et al*.^12^ and Peled *et al*.^13^). These studies suggest that inhibiting CXCR4 might form an important arm of future therapeutic approaches for blood cancer of specific lineages. However, whether CXCR4 inhibition solely mediates AML cells intravasation and mobilization from BM, or BM microenvironment interactions on a wider scale is not known.

To address the questions outlined above, we used intravital microscopy of calvarium BM to study the biology of AML in the BM using the well‐established preclinical model of MLL‐AF9‐driven AML.^14^ We characterized (1) migration of AML cells *in vivo* prior to and following chemotherapy*,* (2) expression of CXCR4 of early infiltrating and chemoresistant cells, and (3) the role of CXCR4 inhibition on the biology of AML within the BM.

## Results

### Heterogeneous *in vivo* migration of AML cells at different disease stages

**Figure 1:**
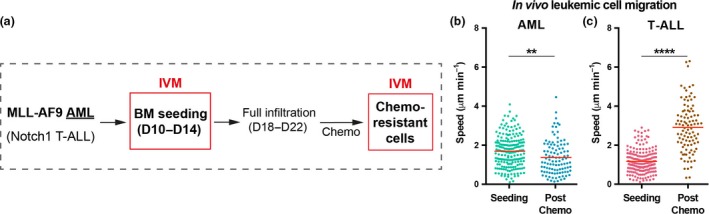
Chemotherapy differentially affects T‐cell acute lymphoblastic leukemia (T‐ALL) and acute myeloid leukemias (AML) cell migration. **(a)** Intravital imaging schedule of mice carrying either T‐ALL or AML blasts at early disease stages, when individual seeding cells could be tracked, and at later stages, when fully infiltrated mice were treated with disease‐specific chemotherapy and single chemoresistant cells could be monitored. **(b, c)** Mean speed of single AML **(b)** and T‐ALL **(c)** blasts located in the BM parenchyma at early disease stages (“Seeding”) and after chemotherapy (“Post‐chemo”). Data obtained from 3 or 4 mice per condition, from two independent experiments for AML, and from two independent experiments for T‐ALL. Plotted data represent semi‐automatic tracking of **(b)** 198 seeding AML cells, 103 chemoresistant AML cells, and **(c)** 187 seeding T‐ALL cells, and 97 chemoresistant T‐ALL cells. It should be noted that seeding single AML cells are significantly faster than seeding T‐ALL cells (*P *<* *0.0001; unpaired *t*‐test).

AML was generated by transducing mTomato^+^ or YFP^+^ granulocyte‐macrophage progenitors with retrovirus encoding the MLL‐AF9 oncogene and T‐ALL was generated by transducing fetal liver cells with DsRed‐Notch‐ICN retrovirus. Preleukemic cells were transplanted into sublethally irradiated recipients. Primary leukemias were then isolated and subsequently transplanted for intravital imaging experiments. We analyzed the motility of single AML and T‐ALL cells during disease establishment, when cells were found either as single, isolated cells or small clusters in the BM (‘seeding’ stage), or following treatment (Figure 1a). We treated mice with chemotherapy regimens adequate for either AML (cytarabine plus an anthracycline) or T‐ALL (dexamethasone/vincristine/l‐asparaginase ‐ DVA). Similar to T‐ALL,^5^ single AML cells were highly dynamic at early disease stages (Figure 1b and Supplementary video 1). Seeding AML cells migrated significantly faster than seeding T‐ALL cells (*P *<* *0.0001), (Figure 1b, c and Supplementary video 2) suggesting that migration is a conserved trait of malignant cells from lymphoid and myeloid origin. Following induction chemotherapy, surviving AML cells did not reside stably in confined areas and were significantly less migratory than cells at seeding stage of disease (Figure 1b). This was in contrast with chemoresistant T‐ALL cells that survived DVA treatment and remained the fastest migratory cell population observed (Figure 1c). These observations highlight the opposing migration characteristics of chemoresistant AML and T‐ALL cells and suggest that leukemic cells arising from different hematopoetic lineages retain a migratory phenotype and are not immotile within the marrow space. Furthermore, the migration phenotype of chemoresistant cells is a phenotype independent of chemotherapy‐induced depletion of healthy hematopoietic cells resulting in increased migration.

### AML *in vivo* cell migration is CXCR4‐independent

**Figure 2:**
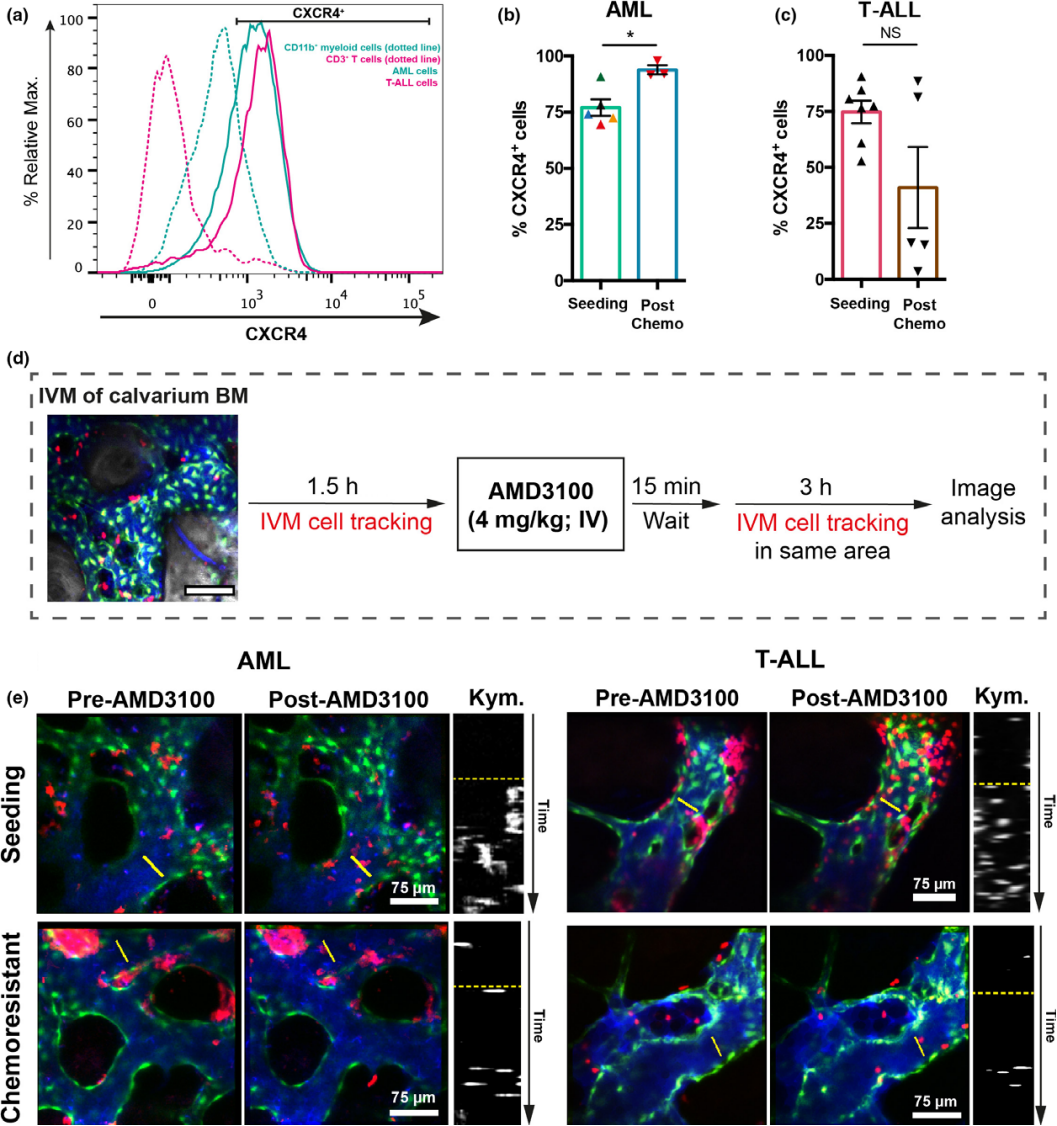
CXCR4 expression and AMD3100 treatment. **(a)** Flow cytometry analysis of CXCR4 expression in acute myeloid leukemias (AML), T‐cell acute lymphoblastic leukemia (T‐ALL), CD3^+^ T and CD11b^+^ myeloid cells. Plots are representative of five independent AML and 4 T‐ALL batches, and bone marrow from three control mice. **(b, c)** Frequency of CXCR4^+^ AML **(b)** and T‐ALL cells **(c)** before and after chemotherapy treatment, assessed by flow cytometry. **(b)** Data obtained by an unpaired *t*‐test from five mice burdened with AML (5 AML batches) untreated and three mice co‐treated with cytarabine and doxorubicin (1 AML batch); each color represents a batch. *P < 0.05 **(c)** Data obtained from seven mice burdened with T‐ALL, untreated, and five mice co‐treated with dexamethasone, vincristine and l‐asparaginase (1 T‐ALL batch). Error bars: mean ± s.e.m. ns, not significant. See the Methods section. **(d)** In each imaging session, several positions within the BM space were selected and timelapsed every 3 min, for 90 min. 15 min after injection of AMD3100, the same positions were timelapsed at the same rate for a further 3 h. **(e)** Maximum intensity projections representative of BM areas showing seeding (four mice analyzed) and chemoresistant (three mice analyzed) AML (left) and seeding (three mice) and chemoresistant (three mice) T‐ALL (right) cell mobilization upon AMD3100 injection. Data are from two independent experiments for AML and from two independent experiments for T‐ALL. Left and middle panels: red, leukemia cells; green, Flk1‐GFP ^+^ endothelial cells; blue, Cy5‐ labeled dextran inside blood vessels. Right panels are kymographs displaying a time projection of the vessel sections highlighted by the yellow lines in the left and middle panels. The dotted yellow lines separate time prior to (top) and following (below) AMD3100 administration.

**Figure 3:**
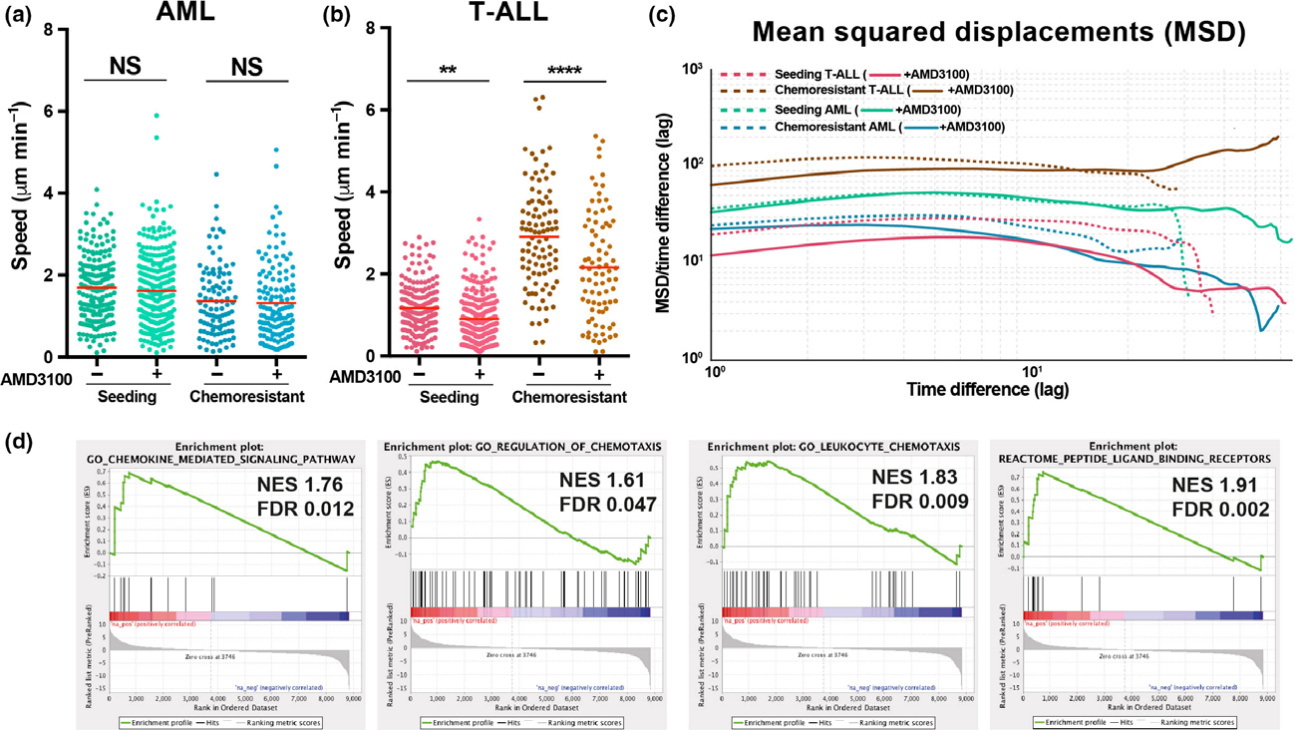
The effect of AMD3100 on *in vivo* leukemic cell migration. **(a, b)** Mean speed of single acute myeloid leukemias (AML) **(a)** and T‐cell acute lymphoblastic leukemia (T‐ALL) **(b)** cells tracked before (−) (same data as in Figure 1b, c) and after (+) exposure to AMD3100. Data obtained for **(a)** AML seeding: *n* = 198 cells (−), *n* = 262 (+) from four mice; AML chemoresistant: *n* = 103 cells (−) and *n* = 134 cells (+) from three mice. Data obtained for **(b)** T‐ALL seeding: *n* = 187 cells (−), *n* = 265 cells (+) from three mice; T‐ALL chemoresistant: *n* = 97 cells (−), *n* = 83 cells (+) from three mice. Data are from three independent experiments for AML and from three independent experiments for T‐ALL (unpaired *t*‐test). **P < 0.01, ****P < 0.0001. See the Methods section. **(c)** Mean‐squared displacements (MSD) of cell tracks were analyzed to characterize the displacements in terms of diffusivity. The log–log plot compares the average squared displacement as a function of the time difference/lag (*x* axis) divided by the lag, for each condition. Chemoresistant T‐ALL cells, upon AMD3100 exposure, changed from diffusive/subdiffusive to superdiffusive motion (red arrow; upward thick brown line). **(d)** Gene Set Enrichment Analysis (GSEA) comparing AML and T‐ALL cells isolated from fully infiltrated BM for genes involved in chemotaxis and chemokine signaling pathways. *n* = 6 mice (T‐ALL) and nine mice (AML), each from one independent experiment.

We,^6^ and others,^2^ have hypothesized that interactions with ligands widely expressed throughout the BM stroma could drive leukemia migration. To investigate this, we measured the expression of CXCR4 on leukemia cells at varying stages of disease. AML blasts expressed higher levels of CXCR4 compared to their healthy myeloid counterparts, similarly to T‐ALL cells relative to healthy lymphoid cells (Figure 2a). Interestingly, the proportion of CXCR4^+^ AML cells increased following chemotherapy (Figure 2b) while it became more variable and overall not significantly different for T‐ALL cells (Figure 2c). This observation is consistent with the hypothesis that AML cells survive in CXCL12‐rich BM niches,^2^ while chemoresistant T‐ALL cells localize stochastically.^5^ To understand the importance of the CXCL‐12/CXCR4 axis, we monitored the short‐term effect of CXCR4 inhibition by performing timelapse intravital microscopy of the same BM areas before and after administering AMD3100 (plerixafor, 4 mg kg^−1^, I.V.; Figure 2d). AMD3100 is a clinically approved CXCR4 antagonist with a median half‐life of 3.6 h^15^ used to mobilize hematopoietic stem and progenitor cells for transplantation. The efficacy of AMD3100 was confirmed by detecting rapid mobilization of both AML and T‐ALL cells from the BM (Figure 2e, Supplementary videos 3, 4), a known effect of CXCR4 antagonism.^8^, ^10^ Interestingly, a significant number of leukemia cells remained within the BM parenchyma. Therefore, we investigated the effect of CXCR4 antagonism on the migration of the remaining nonmobilized, parenchymal AML cells (Figure 3a). Strikingly, the speed of AML cells was not affected by AMD3100, regardless of the disease state (Figure 3a, Supplementary figure 1 and video 1). In contrast, AMD3100 decreased the migration of T‐ALL at both seeding and postchemotherapy disease stages (Figure 3b, Supplementary figures 1, 2 and video 2). The lack of response of AML cells was counterintuitive. To investigate whether CXCR4 inhibition could affect traits of AML migration other than speed, we performed refined analysis of local displacement (Supplementary figure 2) and ability to explore the surrounding BM space (“distinct sites visited” in Supplementary figure 3). These parameters remained unchanged following AMD3100 administration indicating AML cells continued exploring the BM microenvironment highlighting CXCR4 inhibition only affects mobilization of AML cells.

### CXCR4 does not regulate the *in vivo* exploratory behavior of AML cells

**Figure 4:**
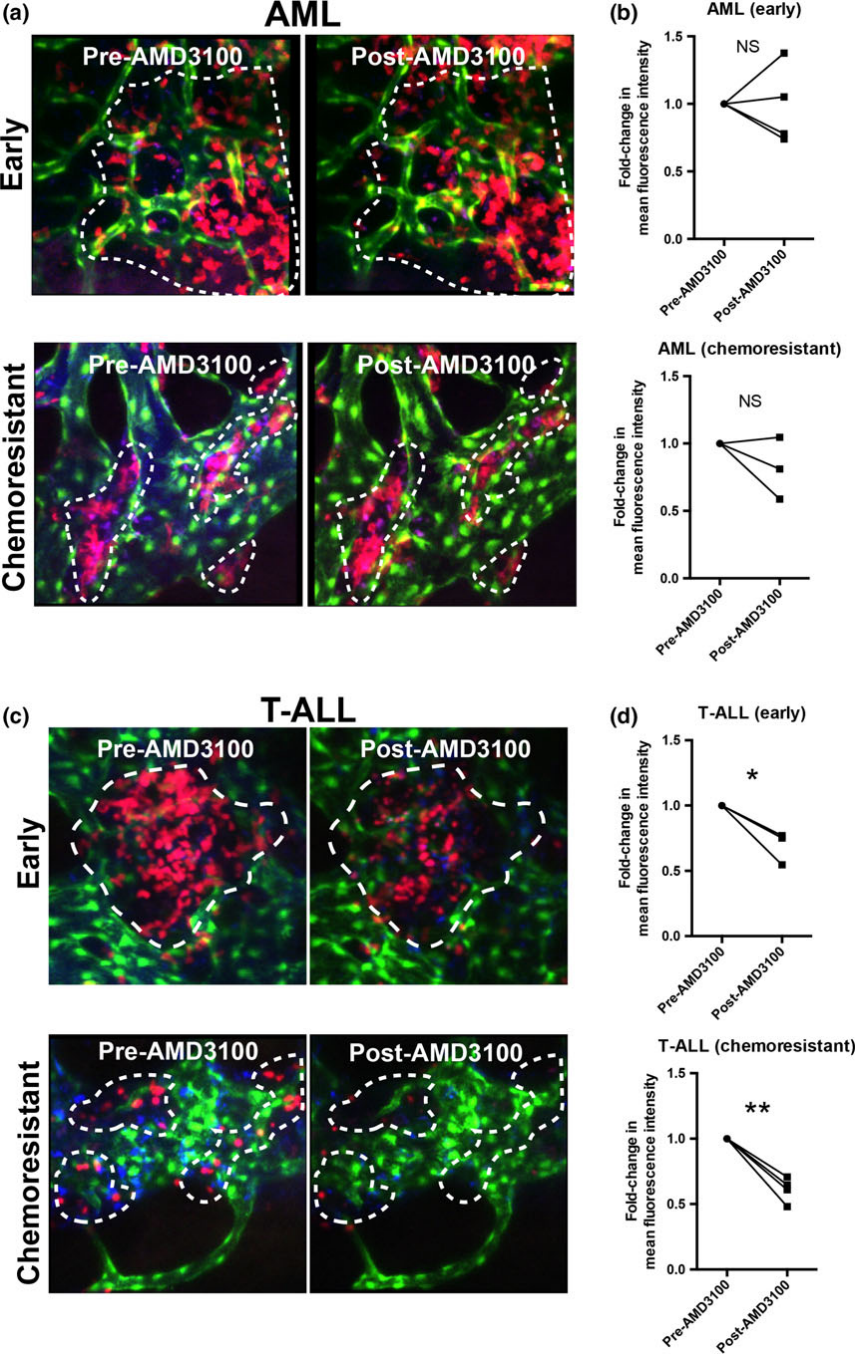
AMD3100 effect on acute myeloid leukemias (AML) and T‐cell acute lymphoblastic leukemia (T‐ALL) cell clusters. Representative maximum projections from intravital microscopy (IVM) three‐dimensional stacks acquired prior to and 180 min following AMD3100 administration, showing the effect of the drug on AML **(a)** and T‐ALL **(c)** cell clusters. CXCR4 antagonism produced **(b)** minimal effect on AML cluster sizes and **(d)** a significant reduction in early colonizing and chemoresistant T‐ALL cell clusters as quantified by paired analysis of mean fluorescence intensity of the same areas before and 180 min after AMD3100 injection. **(a, b)** Data obtained from four mice with early AML infiltration and three mice with chemoresistant AML (paired *t*‐test). Data are from two independent experiments. **(c, d)** Data obtained from three mice with early T‐ALL infiltration and four mice with chemoresistant T‐ALL. Data are from two independent experiments. Red: AML/T‐ALL cells; green: Flk1‐GFP ^+^ endothelial cells; blue: Cy5‐labeled blood vessels. *P < 0.05, **P < 0.01. Paired *t*‐test.

To test the significance of the CXCL12‐CXCR4 axis for parenchymal AML cells within the context of leukemia cell–niche interactions, we characterized the space explored before and after AMD3100 administration by analyzing the time‐averaged mean‐squared displacement of leukemia cell tracks, which indicates how far particle movement deviates from Brownian motion toward ballistic motion (Figure 3c).^16^ These two types of motion can be interpreted as indicators of an explorative/foraging (Brownian), versus environment‐agnostic (ballistic) behavior. Parenchymal AML cells maintained a random‐like diffusive/subdiffusive movement independently of CXCR4 inhibition (Figure 3c). This is an efficient environment‐sampling strategy^17^ and suggests that AML cells do not rely uniquely on CXCR4 signaling to interact with the BM microenvironment. This trait is not common to all leukemia types, because seeding T‐ALL blasts had a less marked downward slope following AMD3100 administration (Figure 3c) and chemoresistant T‐ALL blasts uniquely shifted to a super‐diffusive movement when exposed to AMD3100 (Figure 3c). This suggests that signals allowing retention and interaction with the BM microenvironment were lost by chemoresistant T‐ALL cells upon AMD3100 treatment. We therefore hypothesized that CXCL12 is key to the migratory behavior of T‐ALL, whereas AML relies on additional factors and a more complex crosstalk with the BM microenvironment. To test this, we performed Gene Set Enrichment Analysis of RNAseq data from AML and T‐ALL cells.^18^ This showed that genes involved in chemokine signaling pathways and chemotaxis are enriched in AML cells compared to T‐ALL cells (Figure 3d, Supplementary tables 1–4). Together, track analyses and transcriptomic data suggest that redundancy in chemokine signals might be a feature of AML cell migration, interaction with the BM microenvironment and survival. Consistent with this, we observed minimal AML cell death or reduction in AML cell cluster sizes after AMD3100 treatment (Figure 4a, b and Supplementary video 5) in contrast to T‐ALL (Figure 4c, d and Supplementary video 6).

## Discussion

Here, we show that cell motility is a characteristic feature of both AML and T‐ALL. However, in contrast to T‐ALL, chemoresistant AML cells are less migratory suggesting that they are more engaged with the BM microenvironment. Furthermore, CXCR4 regulates BM retention of both leukemias, but is redundant in fine‐tuning leukemia‐supporting interactions between parenchymal AML cells and the BM microenvironment. In contrast, T‐ALL cells depend on CXCR4 to interact with and survive within the BM parenchyma. The latter observation is consistent with previous reports^8^, ^19^ suggesting that CXCR4 inhibition is a promising avenue for improving T‐ALL treatments. In contrast, our study highlights AML is a more complex disease, able to establish dynamic and redundant interactions with the BM microenvironment, with additional chemokine‐driven pathways likely balancing loss of CXCR4 signaling in parenchymal, nonmobilized AML cells (Supplementary figure 4). Candidates that may be involved in the regulation of AML cell behavior include the receptors CCR1 and CCR2, which are enriched in AML cells (Supplementary tables 1–4). These receptors are also expressed by primary human AML cells^20^ and bind several chemokines, including the strong monocyte chemoattractant CCL2. Furthermore, AML cells themselves express high levels of chemokines^21^ (Supplementary tables 1–4) that may constitute a stimulus for cell migration during disease expansion (positive feedback). Altogether, the poor response to CXCR4 antagonism in AML is consistent with disappointing results from clinical studies and uncertainty about the group of patients that would benefit from this treatment strategy.^13^ While it has been proposed that stronger inhibitors than AMD3100 may improve treatment outcome,^22^, ^23^ our study suggests that the limited effectiveness of CXCR4 antagonists in AML might be partially explained by its mode of action being limited to blast mobilization, while lacking an effect on the behavior of parenchymal AML. This is consistent with the unsatisfactory results obtained by combining CXCR4 inhibition with further mobilization through G‐CSF.^24^ Future studies should investigate factors other than CXCR4 that regulate AML cell migration and/or survival *in vivo* and whether additional interventions may complement chemotherapy and CXCR4 inhibition.

## Methods

The AML and T‐ALL experimental models were as described.^5^, ^14^ Chemotherapy was administered as described.^5^, ^25^ To inhibit CXCR4, mice were i.v. injected with 4 mg kg^−1^ AMD3100 octahydrochloride hydrate (Sigma‐Aldrich, St Louis, MO, USA). Intravital microscopy and image analysis were performed as described.^5^ Data, including track mean speed and cell coordinates, were exported from Imaris (Bitplane, Zürich, Switzerland) for further analysis. Statistical analysis was performed using GraphPad Prism (GraphPad Software Inc., La Jolla, CA, USA) and Python. Further details for all experimental procedures are available as supplementary information, including details of the Gene Set Enrichment Analysis and mathematical analysis.

## References

[1] Dohner H , Weisdorf DJ , Bloomfield CD . Acute myeloid leukemia. N Engl J Med 2015; 373: 1136–1152. 26376137 10.1056/NEJMra1406184

[2] Lane SW , Scadden DT , Gilliland DG . The leukemic stem cell niche: current concepts and therapeutic opportunities. Blood 2009; 114: 1150–1157. 19401558 10.1182/blood-2009-01-202606 PMC2723012

[3] Duarte D , Hawkins ED , Akinduro O , *et al* Inhibition of endosteal vascular niche remodeling rescues hematopoietic stem cell loss in AML. Cell Stem Cell 2018; 22: 64–77.e66 29276143 10.1016/j.stem.2017.11.006 PMC5766835

[4] Ishikawa F , Yoshida S , Saito Y , *et al* Chemotherapy‐resistant human AML stem cells home to and engraft within the bone‐marrow endosteal region. Nat Biotechnol 2007; 25: 1315–1321. 17952057 10.1038/nbt1350

[5] Hawkins ED , Duarte D , Akinduro O , *et al* T‐cell acute leukaemia exhibits dynamic interactions with bone marrow microenvironments. Nature 2016; 538: 518–522. 27750279 10.1038/nature19801 PMC5164929

[6] Duarte D , Hawkins ED , Lo Celso C . The interplay of leukemia cells and the bone marrow microenvironment. Blood 2018; 131: 1507–1511. 29487069 10.1182/blood-2017-12-784132

[7] Rashidi NM , Scott MK , Scherf N , *et al* In vivo time‐lapse imaging shows diverse niche engagement by quiescent and naturally activated hematopoietic stem cells. Blood 2014; 124: 79–83. 24850759 10.1182/blood-2013-10-534859 PMC4125355

[8] Pitt LA , Tikhonova AN , Hu H , *et al* CXCL12‐producing vascular endothelial niches control acute T cell leukemia maintenance. Cancer Cell 2015; 27: 755–768. 26058075 10.1016/j.ccell.2015.05.002 PMC4461838

[9] Zeng Z , Shi YX , Samudio IJ , *et al* Targeting the leukemia microenvironment by CXCR4 inhibition overcomes resistance to kinase inhibitors and chemotherapy in AML. Blood 2009; 113: 6215–6224. 18955566 10.1182/blood-2008-05-158311 PMC2699240

[10] Nervi B , Ramirez P , Rettig MP , *et al* Chemosensitization of acute myeloid leukemia (AML) following mobilization by the CXCR4 antagonist AMD3100. Blood 2009; 113: 6206–6214. 19050309 10.1182/blood-2008-06-162123 PMC2699239

[11] Abraham M , Klein S , Bulvik B , *et al* The CXCR4 inhibitor BL‐8040 induces the apoptosis of AML blasts by downregulating ERK, BCL‐2, MCL‐1 and cyclin‐D1 via altered miR‐15a/16‐1 expression. Leukemia 2017; 31: 2336–2346. 28280274 10.1038/leu.2017.82

[12] Cho BS , Kim HJ , Konopleva M . Targeting the CXCL12/CXCR4 axis in acute myeloid leukemia: from bench to bedside. Korean J Intern Med 2017; 32: 248–257. 28219003 10.3904/kjim.2016.244 PMC5339474

[13] Peled A , Klein S , Beider K , Burger JA , Abraham M . Role of CXCL12 and CXCR4 in the pathogenesis of hematological malignancies. Cytokine 2018; 109: 11–16. 29903571 10.1016/j.cyto.2018.02.020

[14] Krivtsov AV , Twomey D , Feng Z , *et al* Transformation from committed progenitor to leukaemia stem cell initiated by MLL‐AF9. Nature 2006; 442: 818–822. 16862118 10.1038/nature04980

[15] Cashen A , Lopez S , Gao F , *et al* A phase II study of plerixafor (AMD3100) plus G‐CSF for autologous hematopoietic progenitor cell mobilization in patients with Hodgkin lymphoma. Biol Blood Marrow Transplant 2008; 14: 1253–1261. 18940680 10.1016/j.bbmt.2008.08.011

[16] Harris TH , Banigan EJ , Christian DA , *et al* Generalized Levy walks and the role of chemokines in migration of effector CD8 + T cells. Nature 2012; 486: 545–548. 22722867 10.1038/nature11098 PMC3387349

[17] Guigas G , Weiss M . Sampling the cell with anomalous diffusion—the discovery of slowness. Biophys J 2008; 94: 90–94. 17827216 10.1529/biophysj.107.117044 PMC2134854

[18] Waibel M , Vervoort SJ , Kong IY , *et al* Epigenetic targeting of Notch1‐driven transcription using the HDACi panobinostat is a potential therapy against T‐cell acute lymphoblastic leukemia. Leukemia 2018; 32: 237–241. 28914259 10.1038/leu.2017.282

[19] Passaro D , Irigoyen M , Catherinet C , *et al* CXCR4 is required for leukemia‐initiating cell activity in T cell acute lymphoblastic leukemia. Cancer Cell 2015; 27: 769–779. 26058076 10.1016/j.ccell.2015.05.003

[20] Bruserud O , Ryningen A , Olsnes AM , *et al* Subclassification of patients with acute myelogenous leukemia based on chemokine responsiveness and constitutive chemokine release by their leukemic cells. Haematologica 2007; 92: 332–341. 17339182 10.3324/haematol.10148

[21] Mazur G , Wrobel T , Butrym A , Kapelko‐Slowik K , Poreba R , Kuliczkowski K . Increased monocyte chemoattractant protein 1 (MCP‐1/CCL‐2) serum level in acute myeloid leukemia. Neoplasma 2007; 54: 285–289. 17822317

[22] Cho BS , Zeng Z , Mu H , *et al* Antileukemia activity of the novel peptidic CXCR4 antagonist LY2510924 as monotherapy and in combination with chemotherapy. Blood 2015; 126: 222–232. 26031918 10.1182/blood-2015-02-628677 PMC4497963

[23] Borthakur G , Ofran Y , Nagler A , *et al* The peptidic CXCR4 antagonist, BL‐8040, significantly reduces bone marrow immature leukemia progenitors by inducing differentiation, apoptosis and mobilization: results of the dose escalation clinical trial in acute myeloid leukemia. Blood 2015; 126: 2546.

[24] Uy GL , Rettig MP , Stone RM , *et al* A phase 1/2 study of chemosensitization with plerixafor plus G‐CSF in relapsed or refractory acute myeloid leukemia. Blood Cancer J 2017; 7: e542. 28282031 10.1038/bcj.2017.21 PMC5380905

[25] Wunderlich M , Mizukawa B , Chou FS , *et al* AML cells are differentially sensitive to chemotherapy treatment in a human xenograft model. Blood 2013; 121: e90–e97. 23349390 10.1182/blood-2012-10-464677 PMC3606073

