# Supplementary material for: Defining the in vivo characteristics of acute myeloid leukemia cells behavior by intravital imaging
Source: Immunol Cell Biol. 2018 Dec 13;97(2):229–35. doi: 10.1111/imcb.12216 (PMC6446728; doi:10.1111/imcb.12216)
Supplement: Supplementary file 1 [file IMCB-97-229-s001.pdf]

SUPPLEMENTARY ONLINE MATERIAL

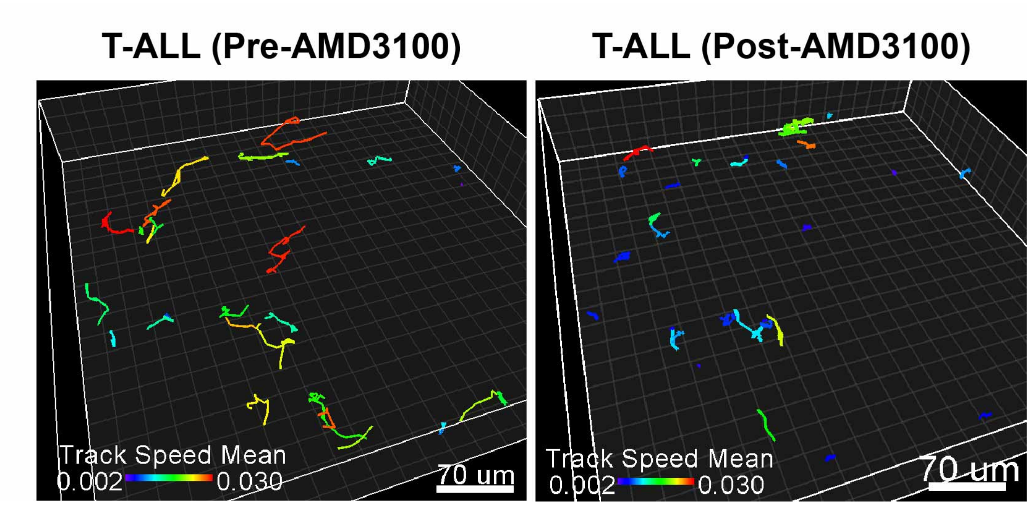

Supplementary figure 1. IVM timelapse analysis of leukemic cells. Representative tracks of seeding T-ALL cells before (1.5 hours) and after (3 hours) exposure to AMD3100.

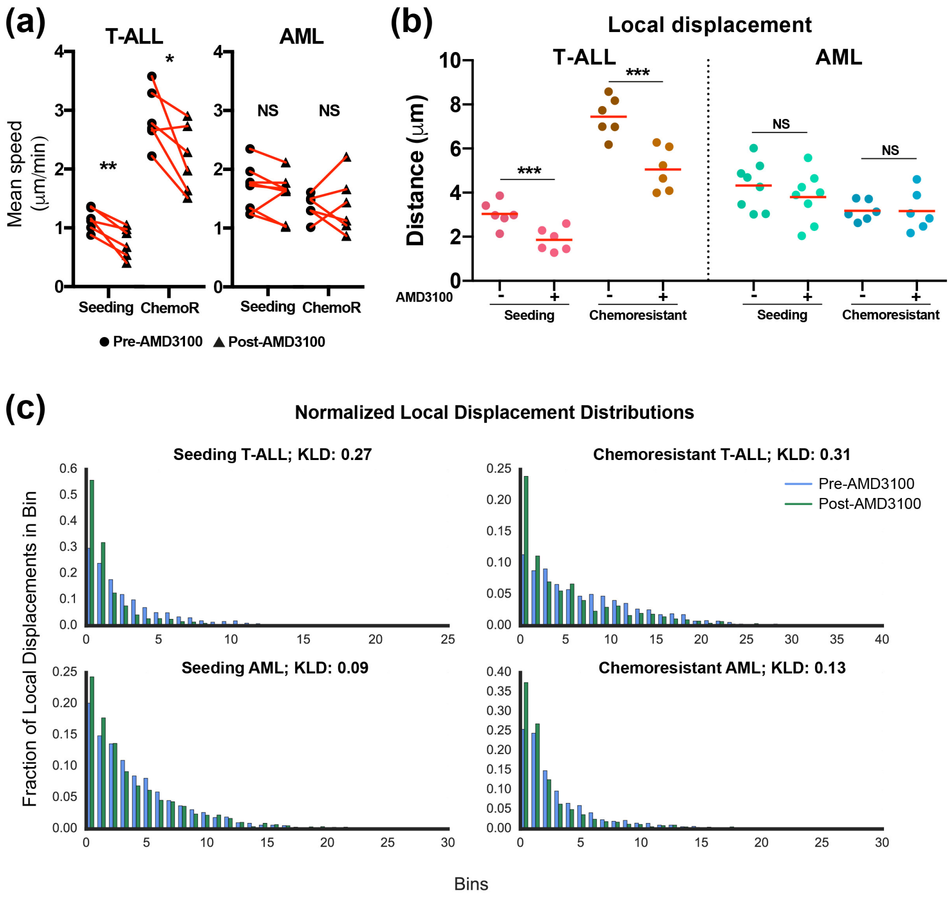

Supplementary figure 2. Local displacement of leukemic cells. (**a**) Paired analysis of mean speed of tracks in the same areas before and after AMD3100 injection. Data obtained as in Figure 3a and b; each dot represents a position. **(b)** AMD3100 significantly diminishes the local cell displacement of seeding and chemoresistant T-ALL cells, but not of AML cells. Shown is the averaged local displacement measured in each of the positions shown in panel A. **(c)** The normalised histograms show the displacement densities (distribution) for each of the conditions, Pre-AMD3100 (blue) and Post-AMD3100 (green). The most striking change in distribution profile is seen for T-ALL cells, where the distribution become less peaked around 0, leading to a reduced slope across bin heights. To quantify the differences between distributions we used Kullback-Liebler divergence (scale [0,1]). The KL divergences confirm the relatively large change in slope in the chemoresistant T-ALL condition, while in other cases the differences are smaller (and marginal for AML cells).

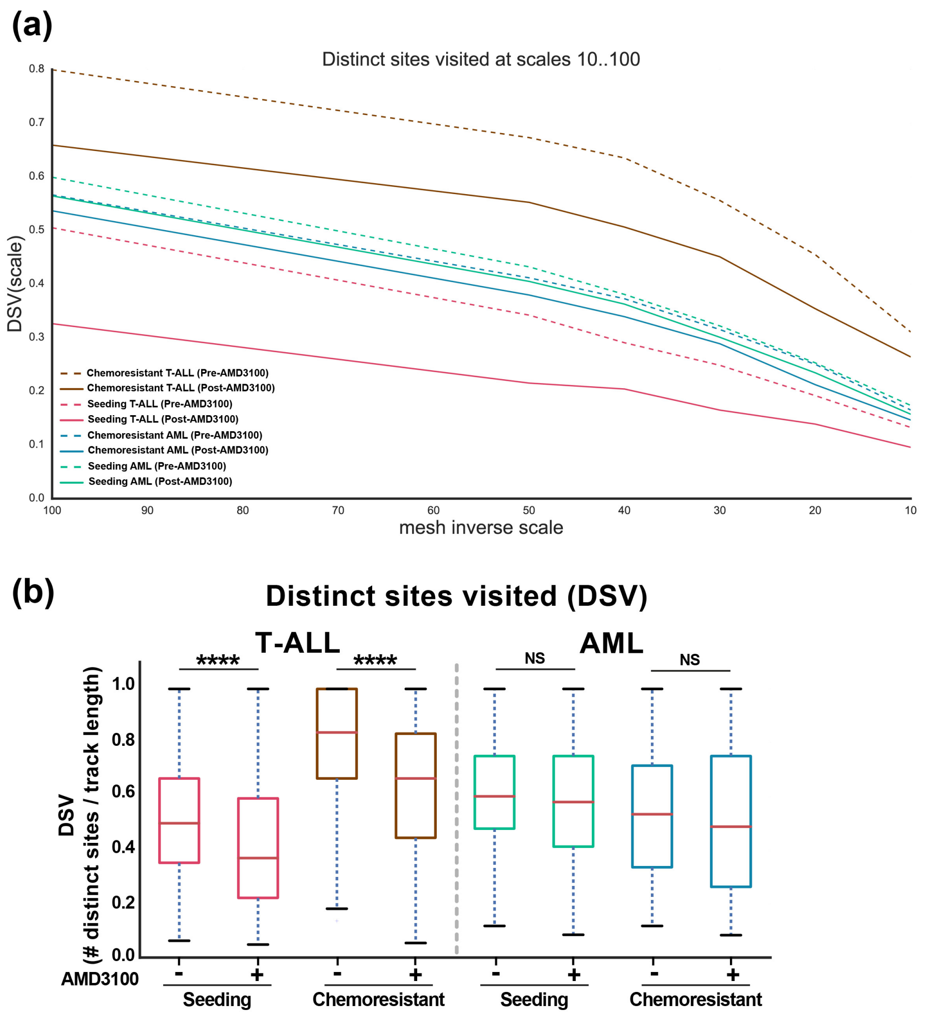

Supplementary figure 3. Distinct sites visited across different scales. To determine how leukemic cells explored the surrounding BM space, a “distinct sites visited" metric was derived by partitioning the volume of each BM position using a range of scales, from coarse to fine-grained, and quantifying how many partitions were visited over time. **(a)** "Scaling behaviour" of distinct sites visited (dsv) resulting from applying the dsv metric across multiple scales. On the x-axis from left (coarse-grained) to right (fine-grained), we plotted dsv (between 0 and 1 on the y-axis, with 0 indicating no new sites apart from initial ones are visited, and 1 all available sites visited over time). The plot shows not only the relative differences between conditions but also the scaling, as we consider track exploration at increasingly fine resolution. For example, the most exploratory condition is the chemoresistant T-ALL (Pre-AMD3100, highest y values). After AMD3100 injection, only seeding and chemoresistant T-ALL cells visited a lower number of sites. **(b)** Treatment-naïve and chemoresistant T-ALL, but not AML, become less exploratory after AMD3100 exposure, as assessed by the number of distinct sites visited. Red lines are median measurements, boxes include the 50% of measurements around the median and whiskers cover the remaining measurements. Shown are the results for mesh size of 100 shown in (a).

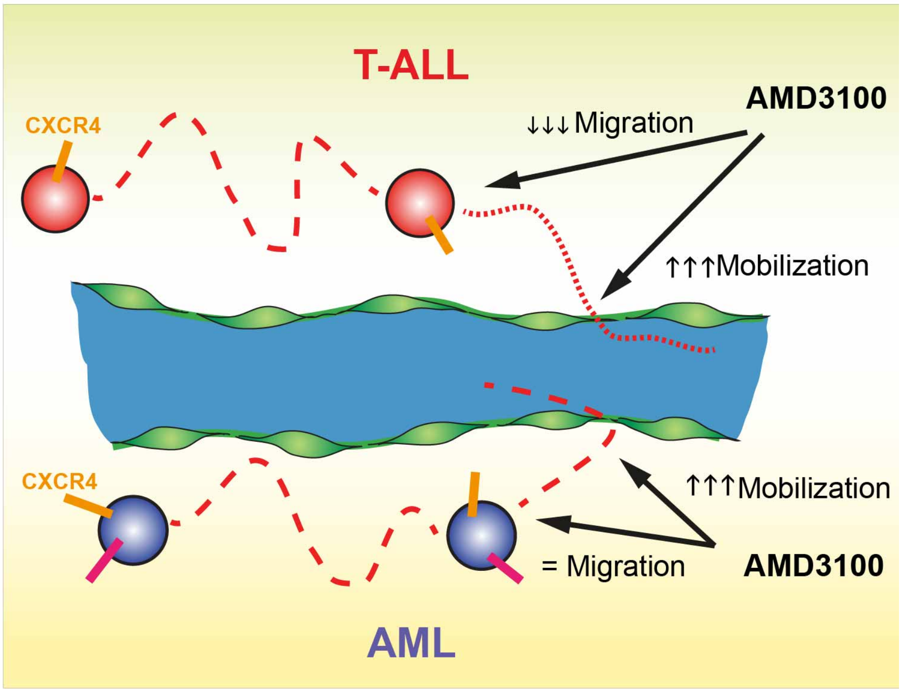

Supplementary figure 4. Proposed model for the effect of CXCR4 inhibition on AML and T-ALL cells. Both T-ALL and AML cells express high levels of CXCR4 at the cell surface level. CXCR4 antagonism can be achieved through the use of the clinically available small molecule AMD3100 or plerixafor. Using IVM, we show that administration of AMD3100 promotes the *in vivo* ousting of both propagating and chemoresistant AML and T-ALL cells from the BM. This is consistent with the knowledge that CXCL12 is a key BM retention and homing signal. In contrast, the motility of leukemia cells from different lineages is differentially impacted by CXCR4 antagonism. While AMD3100 significantly decreases the migration of both propagating and chemoresistant T-ALL cells, it does not affect the movement of AML cells.

Supplementary table 1 – List of enriched genes in the GSEA analysis "*Chemokine mediated signaling pathway"* shown in Figure 3d.

GO_CHEMOKINE_MEDIATED_SIGNALING_PATHWAY				
**PROBE**	**RANK IN GENE LIST**	**RANK METRIC SCORE**	**RUNNING ES**	**CORE ENRICHMENT**
CCL3	203	5,133283615	0,08070138	Yes
CCL18	204	5,133283615	0,18438217	Yes
CCL3L3	205	5,133283615	0,28806296	Yes
CCR1	210	5,105508804	0,39072993	Yes
CCR2	441	3,310226679	0,4315533	Yes
CXCL3	518	3,064232588	0,48484078	Yes
CXCL1	519	3,064232588	0,5467314	Yes
CXCL2	520	3,064232588	0,608622	Yes
CX3CR1	556	2,876471758	0,6627583	Yes
CCL2	738	2,196804285	0,6866398	Yes
CCL15	1551	1,023646235	0,6153975	No
CCL23	1552	1,023646235	0,6360729	No
CCL4	2167	0,641706705	0,5795297	No
CXCR4	2833	0,34236744	0,51116747	No
CIB1	3789	-0,020158617	0,40346956	No
PTK2B	3880	-0,048857506	0,39426845	No
CCR7	8773	-8,328522682	0,008716332	No

Supplementary table 2 – List of enriched genes in the GSEA analysis "*Regulation of chemotaxis"* shown in Figure 3d.

GO_REGULATION_OF_CHEMOTAXIS				
PROBE	RANK IN GENE LIST	RANK METRIC SCORE	RUNNING ES	CORE ENRICHMENT
F7	0	10,15548992	0,07350727	Yes
LBP	87	6,542850971	0,11107731	Yes
ELANE	105	6,242546558	0,1543271	Yes
CCL3	203	5,133283615	0,18044244	Yes
CCR1	210	5,105508804	0,21671413	Yes
IL6R	314	4,255020618	0,23578954	Yes
MET	373	3,76494956	0,2564395	Yes
TIAM1	376	3,715384007	0,2831045	Yes
STX3	383	3,67607832	0,30902973	Yes
PADI2	405	3,520959139	0,3321249	Yes
CCR2	441	3,310226679	0,3521013	Yes
CXCL3	518	3,064232588	0,36563066	Yes
CXCL1	519	3,064232588	0,3878101	Yes
CXCL2	520	3,064232588	0,4099896	Yes
FN1	547	2,937853098	0,42829505	Yes
SEMA4A	549	2,911321402	0,4492539	Yes
PLXND1	727	2,235071898	0,44528607	Yes
CCL2	738	2,196804285	0,46004876	Yes
LYN	790	2,079992771	0,46929944	Yes
RAC2	927	1,760790586	0,46656516	Yes
AIF1	1000	1,647079468	0,47029218	Yes
TGFB1	1189	1,365283847	0,45877668	No
PPM1F	1310	1,235434055	0,45406088	No
LGALS9	1561	1,019455552	0,4329855	No
CORO1B	1645	0,954033256	0,43044415	No
TIRAP	1984	0,748717427	0,3973932	No
CCL4	2167	0,641706705	0,38132322	No
IL16	2348	0,560674846	0,36489433	No
GPSM3	2700	0,394473344	0,32779968	No
ADAM17	2713	0,387390345	0,32923788	No
VEGFA	2749	0,3726601	0,32795164	No
ADAM10	2824	0,34682399	0,32203954	No
CXCR4	2833	0,34236744	0,32360712	No
NCKAP1L	2916	0,308796704	0,31650922	No
CALR	2954	0,298236251	0,31445664	No
MPP1	3000	0,277209342	0,31134138	No
USP14	3529	0,081111714	0,25183287	No

PDGFRB	3602	0,053391952	0,24402447	No
PTK2B	3880	-0,048857506	0,21285067	No
VEGFB	4005	-0,094021827	0,19941786	No
CREB3	4050	-0,106298432	0,1951793	No
CAMK1D	4237	-0,166252315	0,17521262	No
AKT2	4274	-0,176383391	0,17239189	No
SEMA4D	4449	-0,238386244	0,15431313	No
PLXNC1	4904	-0,391598046	0,10547448	No
STX4	5561	-0,616984308	0,03527609	No
SEMA4C	5648	-0,653857231	0,030220525	No
C1QBP	5675	-0,662047982	0,032053296	No
RAC1	5698	-0,668380797	0,03438717	No
PTPRO	6171	-0,871232033	-0,013028522	No
ANO6	6199	-0,887188435	-0,009679963	No
IL1B	6415	-0,977894843	-0,027072532	No
HMGB1	6755	-1,122810721	-0,057529535	No
BMPR2	7053	-1,274315715	-0,082109585	No
SEMA4B	7146	-1,333665133	-0,08292748	No
PTPN2	7205	-1,369705796	-0,079614714	No
PRKD2	7695	-1,791781187	-0,122302204	No
STAP1	7898	-2,014371157	-0,13071294	No
SWAP70	8015	-2,202749252	-0,12797187	No
SMAD3	8322	-3,107149363	-0,1403099	No
NRP1	8411	-3,582346439	-0,12439616	No
CD74	8484	-4,242264748	-0,10188474	No
NOTCH1	8493	-4,333322048	-0,07142991	No
SEMA7A	8691	-7,12221384	-0,042300068	No
CCR7	8773	-8,328522682	0,00876407	No

Supplementary table 3 – List of enriched genes in the GSEA analysis "Leukocyte chemotaxis" shown in Figure 3d.

GO_LEUKOCYTE_CHEMOTAXIS				
PROBE	RANK IN GENE LIST	RANK METRIC SCORE	RUNNING ES	CORE ENRICHMENT
LBP	87	6,542850971	0,049069043	Yes
ITGA1	89	6,481617928	0,10736016	Yes
FCER1G	201	5,146949768	0,1411234	Yes
CCL3	203	5,133283615	0,1872649	Yes
CCL18	204	5,133283615	0,23352006	Yes
CCL3L3	205	5,133283615	0,2797752	Yes
CCR1	210	5,105508804	0,3253255	Yes
TREM1	299	4,395771503	0,35493392	Yes
IL6R	314	4,255020618	0,39168411	Yes
ANXA1	399	3,54687953	0,4140979	Yes
CCR2	441	3,310226679	0,43926618	Yes
CXCL3	518	3,064232588	0,45824012	Yes
VAV3	531	3,025216341	0,48413604	Yes
CX3CR1	556	2,876471758	0,50732785	Yes
CCL2	738	2,196804285	0,5065524	Yes
KIT	855	1,939997077	0,5108501	Yes
SYK	869	1,902852297	0,5265189	Yes
CORO1A	905	1,800390959	0,5387642	Yes
VAV1	1111	1,462034464	0,5286402	Yes
LGALS3	1269	1,27756238	0,5223092	Yes
ITGB2	1327	1,220535874	0,52682924	Yes
FFAR2	1368	1,181957483	0,53293365	Yes
CCL15	1551	1,023646235	0,5214734	Yes
CCL23	1552	1,023646235	0,5306973	Yes
CKLF	1644	0,95429045	0,52895415	Yes
IL17RA	1661	0,946500361	0,53566456	Yes
PIK3CG	1674	0,938457251	0,54275703	Yes
LYST	1729	0,898932397	0,5447201	Yes
CCL4	2167	0,641706705	0,5008377	No
ADAM8	2191	0,63034004	0,5039036	No
IL16	2348	0,560674846	0,4912265	No
PIK3CD	2682	0,407723129	0,4570552	No
VEGFA	2749	0,3726601	0,45291233	No
CXCR4	2833	0,34236744	0,44656447	No
NCKAP1L	2916	0,308796704	0,44002774	No
PIP5K1C	3051	0,25248608	0,42707384	No
NUP85	3234	0,186751097	0,40807244	No

GBF1	3277	0,167535529	0,40480882	No
RPS19	3506	0,08792831	0,3796891	No
PREX1	5548	-0,611841202	0,15324411	No
RAC1	5698	-0,668380797	0,14233303	No
S100A8	5833	-0,727110147	0,13365589	No
PTPRO	6171	-0,871232033	0,10320661	No
ANO6	6199	-0,887188435	0,108132385	No
S100A9	6301	-0,931081355	0,10504363	No
PDE4B	6352	-0,953925967	0,10795683	No
IL1B	6415	-0,977894843	0,10972222	No
PDE4D	6625	-1,056008816	0,09548504	No
HMGB1	6755	-1,122810721	0,090941735	No
SBDS	7046	-1,271273255	0,06943867	No
ITGA9	8662	-6,693085194	-0,053794645	No
CCR7	8773	-8,328522682	0,008750852	No

Supplementary table 4 – List of enriched genes in the GSEA analysis "*Reactome peptide ligand binding receptors"* shown in Figure 3d.

REACTOME_PEPTIDE_LIGAND_BINDING_RECEPTORS				
**PROBE**	**RANK IN GENE LIST**	**RANK METRIC SCORE**	**RUNNING ES**	**CORE ENRICHMENT**
PENK	13	8,547304153	0,13221817	Yes
CCL3	203	5,133283615	0,19111407	Yes
CCL3L3	205	5,133283615	0,27129138	Yes
CCR1	210	5,105508804	0,3506947	Yes
C3	352	3,934672117	0,39627644	Yes
APP	369	3,77812171	0,45355946	Yes
ANXA1	399	3,54687953	0,505754	Yes
CCR2	441	3,310226679	0,55288863	Yes
CXCL3	518	3,064232588	0,5922137	Yes
CXCL1	519	3,064232588	0,64014184	Yes
CXCL2	520	3,064232588	0,68807	Yes
CX3CR1	556	2,876471758	0,7290994	Yes
CCL2	738	2,196804285	0,74297094	Yes
CCL4	2167	0,641706705	0,5913598	No
CXCR4	2833	0,34236744	0,52143747	No
HEBP1	7771	-1,866028905	-0,008239181	No
CCR7	8773	-8,328522682	0,008716368	No

SUPPLEMENTARY VIDEO LEGENDS

Supplementary video 1. *In situ* migration of parenchymal AML cells and the effect of AMD3100. Representative maximum projection of 3D time-lapse data (shown at 10 frames per second) of areas from Flk1-GFP mice acquired every 3min for 90 min prior and 180 min post-AMD3100 administration. Single cells at seeding (first part) and chemoresistant (second part) stages were tracked. Red: AML cells; green: Flk1-GFP$^+$ endothelial cells; blue: Cy5- labelled dextran inside blood vessels. The first frame of the blue channel was copied across the full movie to correct for Cy5 signal bleaching. Upon CXCR4 inhibition, parenchymal cells maintain the same migratory behavior as prior to AMD3100 administration.

Supplementary video 2. *In situ* migration of parenchymal T-ALL cells and the effect of AMD3100. Representative maximum projection of 3D time-lapse data (shown at 10 frames per second) of areas from Flk1-GFP mice acquired every 3min for 90 min prior and 180 min post-AMD3100 administration. Single cells at seeding (first part) and chemoresistant (second part) stages were tracked. Red: T-ALL cells; green: Flk1-GFP$^+$ endothelial cells; blue: Cy5-labelled dextran inside blood vessels. The first frame of the blue channel was copied across the full movie to correct for cy5 signal bleaching. Upon CXCR4 inhibition, parenchymal T-ALL cells are significantly less migratory.

Supplementary video 3. AMD3100 promotes intravasation of AML cells. Representative maximum projection of 3D time-lapse data (shown at 10 frames per second) of areas from Flk1-GFP mice acquired every 3min for 90 min prior and 180 min post-AMD3100 administration. Single cells at seeding (first part) and chemoresistant (second part) stages were tracked. Red: AML cells; green: Flk1-GFP$^+$ endothelial cells; blue: Cy5- labelled dextran inside blood vessels. The first frame of the blue channel was copied across the full movie to correct for cy5 signal bleaching. Upon CXCR4 inhibition, mobilized AML cells are visible inside vessels. Parenchymal cells maintain the same migratory behavior as prior to AMD3100 administration.

Supplementary video 4. Seeding and chemoresistant T-ALL cell mobilization. Representative maximum projection of 3D time-lapse data (shown at 10 frames per second) of areas from Flk1-GFP mice acquired every 3min for 90 min prior and 180 min post-AMD3100 administration. Single cells at seeding (first part) and chemoresistant (second part) stages were tracked. Red: T-ALL cells; green: Flk1-GFP$^+$ endothelial cells; blue: Cy5-labelled dextran inside blood vessels. The first frame of the blue channel was copied across the full movie to correct for cy5 signal bleaching. Upon CXCR4 inhibition, mobilized T-ALL cells are visible inside vessels.

Supplementary video 5. AMD3100 induces little AML cell death and minimal impact on cell clusters. Representative maximum projection of 3D time-lapse data (shown at 10 frames per second) of areas from Flk1-GFP mice acquired every 3min for 90 min prior and 180 min post-AMD3100 administration. Cell clusters at seeding (first part) and chemoresistant (second part) stages are marginally affected by AMD3100. Red: AML cells; green: Flk1-GFP$^+$ endothelial cells; blue: Cy5-labelled dextran inside blood vessels. The first frame of the blue channel was copied across the full movie to correct for cy5 signal bleaching. Arrows point at intravasating cells.

Supplementary video 6. AMD3100 induces T-ALL cell death, contributing to dramatic reduction in cell cluster sizes. Representative maximum projection of 3D time-lapse data (shown at 10 frames per second) of areas from Flk1-GFP mice acquired every 3min for 90 min prior and 180 min post-AMD3100 administration. Single cells at seeding (first part) and chemoresistant (second part) stages were tracked. Red: T-ALL cells; green: Flk1-GFP$^+$ endothelial cells; blue: Cy5-labelled dextran inside blood vessels. The first frame of the blue channel was copied across the full movie to correct for cy5 signal bleaching. Clusters of early colonizing (first part) and chemoresistant (second part) T-ALL cells are significantly reduced upon injection of AMD3100. Arrows point at intravasating cells. Cell death caused by AMD3100 is frequent and evidenced by reduction of the cell size and disappearance of fluorescent red signal over time (arrowheads and circles).

METHODS

Mice

All animal work was in accordance with the animal ethics committee at Imperial College London, UK and UK Home Office regulations (ASPA 1986). Flk1-GFP mice were a gift from Alexander Medvinsky (University of Edinburgh) [1], PU1-YFP were a gift from Claus Nerlov (University of Oxford) [2]. C57Bl/6 mice were from Harlan UK Ltd.

T-ALL experimental model

As described in[3, 4]. Briefly, secondary 10,000-50,000 thawed, Ficoll purified Notch DsRed^{+} T-ALL blasts were injected into sub-lethally irradiated tertiary recipients. Early colonization of the BM was detected between day 8 to day 12 post-transplantation. Full infiltration of the BM was observed between day 18 and day 20 post-transplantation and confirmed by sampling of peripheral blood.

AML experimental model

As described in [5]. Briefly, mTomato^{+} or PU.1-YFP^{+} granulocyte monocyte progenitors (GMPs) were sorted from mT/mG or PU.1-YFP mice, transduced with pMSCV-MLL-AF9-GFP-based retroviruses as described in [3] and transplanted into sub-lethally irradiated mice. After 8 weeks post-transplantation, recipient mice developed leukemia and BM and spleen blasts were harvested and stored. 100,000 primary mTomato^{+} or PU.1-YFP^{+} blasts were injected into non-conditioned recipients. Early colonization of the BM was detected between day 10 to day 14 post-transplantation. Full infiltration of the BM was observed between day 20 and day 22 post-transplantation and confirmed by sampling of peripheral blood.

Therapies administration

To model chemoresistance, drugs commonly administered in the treatment regimens of T-ALL and AML patients were used in our mouse models.

Mice burdened with T-ALL were treated daily, for 3 days, with i.v. 15 mg kg^{-1} dexamethasone sodium phosphate (Sellekchem, MA), 0.15 mg kg^{-1} vincristine (Sigma) and 1,000 IU kg^{-1} l-asparaginase (medac; obtained from the Imperial College Healthcare NHS Trust Pharmacy) [4].

Mice burdened with AML were treated daily i.v. with 100mg kg^{-1} cytarabine (Ara-C) I.V. for 5 days and 3mg kg^{-1} doxorubicin (Doxo) for 3 days. Ara-C was co-delivered with Doxo on days 1 to 3 and alone on days 4 and 5, similarly to induction chemotherapy used in AML patients [6]. Both Ara-C and Doxo were obtained from the Imperial College Healthcare NHS Trust Pharmacy.

For CXCR4 inhibition experiments, mice were i.v. injected with 4mg kg^{-1} AMD3100 octahydrochloride hydrate (Sigma-Aldrich).

Flow cytometry

To obtain a single cell suspension of bone marrow cells, bones were crushed, centrifuged at 500g for 5min, resuspended in PBS with 2% fetal bovine serum, filtered through a 40μm strainer and stained. The following fluorochrome-conjugated or biotinilated primary antibodies specific to mouse were used: CD3e (145-2C11), CD4 (GK1.5), CD8a (53-6.7), Ter119 (TER119), B220 (RA3-6B2), Ly6G (RB-68C5), CD11b (M1/70) all from Biolegend and CXCR4 (2B11) from eBiosciences. Streptavidin Pacific Orange (Invitrogen) was used for secondary staining. Dead cells

were excluded using 4,6-diamidino-2-phenylindole (DAPI, Invitrogen). Samples were acquired in a LSR Fortessa and analysed with FlowJo (Tree Star).

Intravital microscopy
As described in [4]. Briefly, we used a Zeiss LSM 780 upright confocal microscope fitted with Argon 488nm, HeNe 561 and 633nm lasers and a tunable infrared multiphoton laser (Spectraphysics Mai Tai DeepSee 690-1040). A W Plan-Apochromat ×20 DIC water immersion lens (1.0 N.A) was used. Second harmonic generation (SHG) signal was used to detect bone collagen. SHG signal (not shown for simplicity) was acquired through excitation at 870nm and detection with an external non-descanned detector (NDD) (<495nm). GFP and YFP signals were excited at 488nm, mTomato at 561nm and Cy5 at 633nm; GFP, YFP, mTomato and YFP signals were detected using internal detectors. Blood vessels were labeled with 80μl of 8mg ml^{-1} 500kD Cy5-Dextran (Nanocs, MA).

RNA sequencing and analysis
RNA-seq was performed as described previously [7]. Total RNA was extracted using RNeasy® Mini Kit (Qiagen, Hilden, Germany). The extracted RNA was analysed on the Agilent 4200 Tapestation prior to library preparation. High quality RNA with RIN values greater than 9 was used for downstream application. 3'mRNA-sequencing libraries were prepared from 100ng of total RNA using the QuantSeq 3' mRNA-Seq Library Prep Kit (Lexogen) according to the manufacturers instructions and sequenced on a NextSeq 500 (Illumina). The single-end 75bp were demultiplexed using CASAVAv1.8.2 and Cutadapt (v1.9) was used for read trimming. The trimmed reads were subsequently mapped to the mouse genome (mm10) using HISAT2. FeatureCounts was used for read counting [8] after which differential gene expression analysis was performed using Voom-LIMMA packages [9]. Data for RNA-seq for AML and T-ALL samples can be accessed through Gene Expression Omnibus (GEO) with the accession numbers GSE105159 and GSE102757 respectively. GSEA2-2.2.2 was used for Gene set enrichment analysis (GSEA) [10, 11].

Image analysis
Imaging data was exported as .lsm files and pre-processed in Fiji/ImageJ. To correct for 3D drift over time, movies of the same area acquired separately were concatenated and registered[26] using the stromal signal (e.g. Flk1-GFP^+) as the reference channel. Imaris (Bitplane, Switzerland) was used to detect the leukemia cells and create either "spots" or "surfaces"; semi-automatic cell tracking was done using built-in algorithms that were manually supervised. Data, including track mean speed and cell coordinates were exported for further analysis.

Mathematical analysis
IMARIS track data in (time, x, y, z) format were processed using Python (version 3.5.2) scientific and data analysis libraries (Pandas, Numpy, Scipy and others from the Anaconda distribution version 2.5.0). Time-interpolation was used (assuming constant speed) to fill gaps in track data arising from various tracking anomalies. Tracks were post-processed and augmented with derived observables given initial 4D data points to produce an extended track dataset. Each data point in this dataset was attributed with both numeric observables and metadata for the *condition*, *stage* and *position*. The **Kullback-Liebler divergences** (KLD) have been reported as a [0,1]-scaled measure of the significance of the differences between displacement distributions before and after

treatment. Values close to 1 indicate that the distributions are very different while values close to 0 indicate that they are not.

A **distinct sites visited** metric was derived by partitioning the position volume on a number of scales a_n. We counted the number of unique volume elements or "sites" at different scales that were visited by individual tracks.

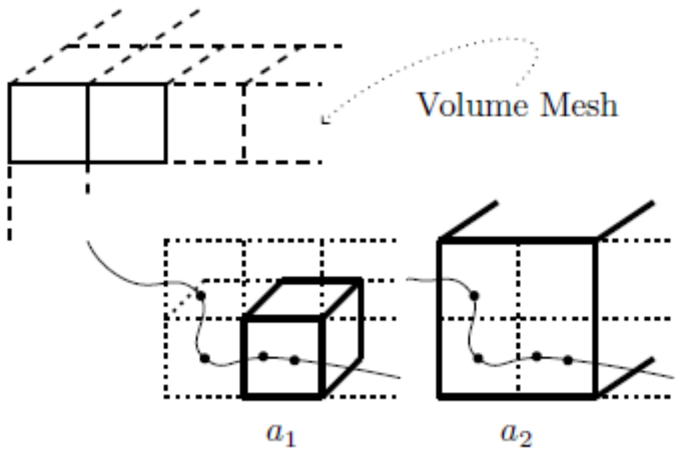

For a given track of length *n*, by dividing the distinct number of sites it visits by it's length *n*, we produce a number between 0 and 1. Unity corresponds to a track visiting a new site (at a particular scale) with each observed jump. We can consider the "scaling behavior" by plotting the number of distinct sites visited as a function of the scale parameter. This method is similar to "box counting" when measuring the fractal dimension of a curve in space. We compared distinct site visited values at the coarse and fine-grained scale. Two-way ANOVA *P*-values indicated statistically significant differences within conditions and between stages for T-ALL conditions allowing us to contrast how the volume was explored.

We studied the **ensemble time-averaged mean squared displacement** as a function of lag for each condition. This is defined as the average over all time lags, over all displacements within an experiment set;

$$\Delta^2(\tau) = \frac{1}{M} \sum_{t=1}^{T_{j-\tau}} ||\boldsymbol{p}(\boldsymbol{t}+\boldsymbol{\tau}) - p(t)||^2$$

Mean squared displacement provides statistical tests relating to particle movement. In particular it provides a test for deviations from "random" linear diffusion. We observed subdiffusive behaviour in most cases with the exception of post-chemotherapy T-ALL post-AMD3100, which showed a transition to superdiffusive.

Statistical analysis

Data was processed using GraphPad Prism (GraphPad Software Inc.) and Python. Group means were compared using the unpaired Student's *t*-test. For multiple comparisons, ANOVA with post-hoc Bonferroni or Tukey test was used. Differences were considered significant (*) whenever $P<0.05$; (**) is used for $P<0.01$, (***) for $P<0.001$ and (****) for $P<0.0001$

REFERENCES

1. Xu Y, Yuan L, Mak J, et al. Neuropilin-2 mediates VEGF-C-induced lymphatic sprouting together with VEGFR3. *J Cell Biol* 2010; **188**: 115-130.
2. Kirstetter P, Anderson K, Porse BT, Jacobsen SE, Nerlov C. Activation of the canonical Wnt pathway leads to loss of hematopoietic stem cell repopulation and multilineage differentiation block. *Nat Immunol* 2006; **7**: 1048-1056.
3. Hawkins ED, Oliaro J, Ramsbottom KM, et al. Lethal giant larvae 1 tumour suppressor activity is not conserved in models of mammalian T and B cell leukaemia. *PLoS One* 2014; **9**: e87376.
4. Hawkins ED, Duarte D, Akinduro O, et al. T-cell acute leukaemia exhibits dynamic interactions with bone marrow microenvironments. *Nature* 2016; **538**: 518-522.
5. Krivtsov AV, Twomey D, Feng Z, et al. Transformation from committed progenitor to leukaemia stem cell initiated by MLL-AF9. *Nature* 2006; **442**: 818-822.
6. Wunderlich M, Mizukawa B, Chou FS, et al. AML cells are differentially sensitive to chemotherapy treatment in a human xenograft model. *Blood* 2013; **121**: e90-97.
7. Waibel M, Vervoort SJ, Kong IY, et al. Epigenetic targeting of Notch1-driven transcription using the HDACi panobinostat is a potential therapy against T-cell acute lymphoblastic leukemia. *Leukemia* 2017; e-pub ahead of print Sep 15;10.1038/leu.2017.282.
8. Liao Y, Smyth GK, Shi W. featureCounts: an efficient general purpose program for assigning sequence reads to genomic features. *Bioinformatics* 2014; **30**: 923-930.
9. Law CW, Chen Y, Shi W, Smyth GK. voom: Precision weights unlock linear model analysis tools for RNA-seq read counts. *Genome Biol* 2014; **15**: R29.
10. Subramanian A, Tamayo P, Mootha VK, et al. Gene set enrichment analysis: a knowledge-based approach for interpreting genome-wide expression profiles. *Proc Natl Acad Sci U S A* 2005; **102**: 15545-15550.
11. Liberzon A, Birger C, Thorvaldsdottir H, Ghandi M, Mesirov JP, Tamayo P. The Molecular Signatures Database (MSigDB) hallmark gene set collection. *Cell Syst* 2015; **1**: 417-425.
